# HIV Prevalence and Risk Factors Among Young Men Who Have Sex With Men in Southwest China: Cross-sectional Questionnaire Study

**DOI:** 10.2196/37344

**Published:** 2023-01-11

**Authors:** Liping Song, Xiangyuan Yu, Bing Su, Wen Kui Geng, Guanghua Lan, Xiangjun Zhang

**Affiliations:** 1 School of Public Health Guilin Medical University Guilin China; 2 Department of Clinical Psychology Qingdao Mental Health Center Qingdao China; 3 Guangxi Zhuang Autonomous Region Health and Family Planning Commission Nanning China; 4 Guangxi Zhuang Autonomous Region Center for Disease Prevention and Control Nanning China; 5 Department of Clinical Pharmacy and Translational Science College of Pharmacy University of Tennessee Health Science Center Memphis, TN United States

**Keywords:** HIV, young men who have sex with men, syphilis, sexually transmitted infection, ethnic minority

## Abstract

**Background:**

Previous studies showed an increase in HIV prevalence among young men who have sex with men aged 25 years or younger in China.

**Objective:**

This study aimed to assess HIV prevalence and associated factors among young men who have sex with men in the Guangxi Zhuang Autonomous Region.

**Methods:**

This study was conducted in 4 cities (Guilin, Liuzhou, Beihai, and Nanning) in the Guangxi Zhuang Autonomous Region between June 2014 and May 2016. Participants were reached through web-based and site recruitment approaches. Laboratory tests were performed to detect HIV and syphilis infections. A self-administered questionnaire was used to collect data from 632 eligible young men who have sex with men.

**Results:**

The prevalence of HIV and syphilis was 9.3% (59/632) and 11.4% (72/632), respectively. Multivariable logistic analysis showed that ethnic minority (adjusted odds ratio [AOR] for Han Chinese vs other minorities 0.28, 95% CI 0.11-0.71, *P*=.007), receptive sexual positioning in the past 6 months (AOR 2.94, 95% CI 1.32-6.53, *P*=.008), current syphilis infection (AOR for individuals without vs those with infection 0.38, 95% CI 0.19-0.75, *P*=.005), inconsistent condom use in the past 6 months (AOR 1.91, 95% CI 1.06-3.45, *P*=.03), and psychotropic drug use before last anal intercourse (AOR 16.70, 95% CI 2.34-119.18, *P*=.005) were independently associated with HIV infection.

**Conclusions:**

There is an urgent need to scale up HIV and syphilis interventions in young men who have sex with men. Some subgroups might need specific attention for HIV prevention, including ethnic minority men, individuals with a history of sexually transmitted infections, and individuals who have been engaging in receptive anal sex.

## Introduction

Men who have sex with men are one of the key and most vulnerable populations in the HIV epidemic [1]. The homosexual transmission rates of annually reported HIV cases in China increased from 2.5% in 2005 to 25.8% in 2014 [2,3], and the new cases of HIV infection among men who have sex with men increased from 487 in 2006 to over 30,000 in 2015 [4]. Epidemiological studies have also demonstrated that HIV prevalence among men who have sex with men has been rising in many Chinese cities in recent years [5-9]. For example, HIV prevalence among men who have sex with men in the capital city of Beijing increased from 0.4% in 2007 to 9.9% in 2010 [10-12] and doubled from 8.5% in 2008 to 16.8% in 2009 in Chongqing in southwest China [13-15]. Young men who have sex with men who are aged 25 years or younger are a subgroup of particular concern as they usually have high HIV prevalence [16] and incidence [17] due to unprotected anal intercourse [18-20] and multiple sexual partnerships [19,20]. One study investigated HIV and syphilis infections among young men who have sex with men who were 16-25 years old in 4 cities in China and found the prevalence of HIV and syphilis to be 6.7% and 8.3%, respectively, in 2008 [16]. Using a prospective cohort design, 1 study reported an HIV incidence rate of 6.7 per 100 person-years among young men who have sex with men in urban areas of China in 2010 [17]. Factors that were associated with young men who have sex with men’s HIV infection in previous literature included nonstudent status, low HIV transmission knowledge, and syphilis infection [16,17]. Further, a meta-analysis study reported rates of having unprotected anal intercourse and having multiple sexual partners in the last 6 months for young men who have sex with men were as high as 65.2% and 58.2%, respectively [20]. Although particular attention needs to be paid to HIV epidemiology and behavioral intervention in young men who have sex with men, the previous literature relatively lacks current data and evidence to better understand the behavioral characteristics of young men who have sex with men in relation to HIV infection.

The previous literature reported some factors that could explain the high HIV prevalence and incidence in the young men who have sex with men group. Men who have sex with men commonly face stigma issues, which prevent them from disclosing of their sexual orientation in Chinese culture [21,22]. Therefore, it was very hard to reach out to them for HIV prevention and intervention purposes [22,23], thus, HIV risk behaviors remained high, such as unprotected anal intercourse among Chinese men who have sex with men [23]. Therefore, young men who have sex with men showed a higher prevalence of recent HIV infection and a higher HIV incidence than men who have sex with men, who are aged 25 years and older [24].

The Guangxi Zhuang Autonomous Region, which is located in southwest China, has a large proportion of minority groups. Ethnic minorities accounted for 37.5% of the total population; of them, the Zhuang ethnicity accounted for 31.4% of the total population in 2020 [25]. Moreover, Guangxi is one of the 6 provinces and autonomous regions with the highest HIV prevalence in China. Historically, injection drug use was the primary cause of the HIV epidemic in Guangxi [26]. Furthermore, recent surveillance data showed that there has been a growing HIV prevalence among young men who have sex with men aged 25 years and younger [26]. To better understand the HIV epidemic among young men who have sex with men with the goal of designing appropriate interventions, we conducted a cross-sectional study to characterize the severity of the HIV epidemic and risk factors among young men who have sex with men in Guangxi Zhuang Autonomous Region.

## Methods

### Study Design and Participants

This cross-sectional study was conducted in 4 cities (Guilin, Liuzhou, Beihai, and Nanning) in the Guangxi Zhuang Autonomous Region between June 2014 and May 2016. The eligibility criteria for participation included being a male; having self-reported anal sex with men in the past year; being aged 18 to 25 years; and being willing and able to provide consent for participation in this study. Participants were recruited by staff from the Centers for Disease Control and Prevention (CDC) in 4 cities through web-based or site recruitment. We also used a snowball sampling procedure. Research staff who had experience working with men who have sex with men communities posted study recruitment information through commonly used social media platforms for men who have sex with men, such as QQ and WeChat. They also visited men who have sex with men gathering venues periodically to distribute study flyers and recruit participants, such as wine bars, dance clubs, coffee or tea shops, bath, sauna, massage, or pedicure rooms, and parks. The recruitment materials had the study information and local clinics’ location, phone number, and office hours. Potential participants could contact or visit the clinics to learn more about the study and participate in it. Participants were encouraged to distribute study recruitment flyers to their social networks.

### Data Collection

A self-administered structured questionnaire was used anonymously to collect data by research staff from the CDC in 4 cities. The questionnaire collected the following data: social-demographics (age, marital status, ethnicity, education, residence, and occupation), sexual behaviors (condom use in the last anal intercourse and in the past 6 months, number of anal sex and sexual positioning—insertive, receptive, or versatile—in the past 6 months, history of drug use and intravenous drug use, commercial sex, history of sexually transmitted disease [STD] in the past 12 months), etc. A written informed consent form was signed by the participants at recruitment. Each participant received 50 Yuan RMB (approximately US $8) upon completion of the survey.

### HIV-Related Knowledge

We also assessed HIV-related knowledge using a scale of 8 questions, and each question has 3 options (true, false, and unsure). Sample questions were “Can an HIV-infected person be recognized in appearance?” “Would mosquito bites spread HIV?” “Would a person be infected by eating together with an HIV-infected person?” and “Can a person reduce the risk of HIV transmission by using condoms correctly in every sexual behavior?” Participants got 1 point for each correct answer and 0 points otherwise. The total score for this scale ranged from 0 to 8, with a score of ≥6 indicating participants have good HIV knowledge [27]. The Cronbach for the scale reliability was .731, which indicates the scale has acceptable internal consistency.

### Serological Tests

Venous blood samples were collected from all participants and tested for HIV and syphilis. HIV screening was conducted using an enzyme-linked immunosorbent assay (ELISA, Vironostika HIV-1 Microelisa System; BioMe´rieux), and a positive case was confirmed by the HIV-1/2 Western blot assay (HIV Blot 2.2 WB; Genelabs Diagnostics). Syphilis tests were conducted by ELISA and rapid plasma regain testing (diagnosis test; Shanghai Kehua, China). A participant who had positive results for both ELISA and rapid plasma regain testing was considered to have a current syphilis infection.

### Statistical Analysis

SPSS (version 19.0; SPSS Inc) was used for the data analyses. We calculated means and SDs for continuous variables and frequencies (%) for categorical variables. Chi-square tests were conducted to assess the associations between HIV infection and categorical independent variables. We conducted stepwise multivariate logistic regression analyses to identify factors that were related to HIV infection [9]. The multivariable analysis model included marginally significant variables with a value of *P*≤.05 in the bivariate analysis (chi-square test). The threshold for statistical significance was set at *P*≤.05.

### Ethical Considerations

Ethical approval was obtained for this study from the institutional review board of the Ethics Committee at Guilin Medical University on March 1, 2013. A signed written informed consent form was obtained from all participants prior to their participation.

## Results

Among the 651 men who have sex with men who enrolled in this study, 632 were eligible young men who have sex with men, and 19 participants were aged beyond the range of 18 to 25 years. Therefore, we only included 632 participants in the final analyses. 

Table 1 presents the sociodemographic characteristics of the study sample. Participants had an average age of 22 years and 70.6% (446/632) of them were of Han ethnicity and 23.4% (148/632) were of Zhuang ethnicity. More than half of the participants had college-associated or higher education (367/632, 58.1%). Most participants (27/632, 95%) were unmarried, and 73.9% (467/632) of them have been living in Guangxi for more than 2 years. About one-third of the participants (238/632, 37.7%) were students. Moreover, approximately 6% (38/632) of participants had a history of an STD in the past 12 months. A few participants had a history of drug use (11/632, 2%) and psychotropic drug use before the last anal intercourse in the past 6 months (5/632, 1%). Regarding sexual behaviors, for anal sexual positioning in the past 6 months, 39.2% (248/632) of participants reported insertive, 33.9% (214/632) reported receptive, and 26.9% (170/632) reported versatile positioning. A majority of participants used a condom in the last anal intercourse (414/632, 65.5%), and more than half of the participants (325/632, 51.4%) used a condom every time in an anal intercourse in the past 6 months. Less than one-third (191/632, 30.2%) of participants engaged in anal intercourse twice or more in the last week. Furthermore, some participants had commercial sex with a male (106/632, 16.8%), sex with a female (70/632, 11%), or were currently syphilis infected (72/632, 11%). Only 7% (43/632) of the participants received less than 6 points on the HIV knowledge scale.

**Table 1:** Demographic characteristics, sexual behaviors, and HIV knowledge by HIV infection among young men who have sex with men in Guangxi, China between 2014 and 2015 (N=632).

Variables			Total, n (%)		HIV infection, n (%)					Chi-square (*df*)			*P* value
					Yes		No						
**Age (years)**													
	18-20	173 (27.4)		17 (9.8)		156 (90.2)		0.068 (1)			.79		
	21-25	459 (72.6)		42 (9.2)		417 (90.8)		N/A^a^			N/A		
**Ethnicity**													
	Han	446 (70.6)		30 (6.7)		416 (93.3)		12.639 (2)			.002		
	Zhuang	148 (23.4)		22 (14.9)		126 (85.1)		N/A			N/A		
	Others	38 (6.0)		7 (18.4)		31 (81.6)		N/A			N/A		
**Education**													
	High school or associated level or below	265 (41.9)		30 (11.3)		235 (88.7)		2.125 (1)			.15		
	College associated level or above	367 (58.1)		29 (7.9)		338 (92.1)		N/A			N/A		
**Marital status with female**													
	Unmarried	597 (94.5)		56 (9.4)		541 (90.6)		0.513 (2)			.773^b^		
	Married	27 (4.3)		2 (7.4)		25 (92.6)		N/A			N/A		
	Others^c^	8 (1.3)		1 (1.3)		7 (87.5)		N/A			N/A		
**Living duration in Guangxi (years)**													
	2 or less	165 (26.1)		18 (10.9)		147 (89.1)		0.653 (1)			.42		
	>2	467 (73.9)		41 (8.8)		426 (91.2)		N/A			N/A		
**Occupation**													
	Student	238 (37.7)		25 (10.5)		213 (89.5)		0.616 (1)			.43		
	Nonstudent	394 (62.3)		34 (8.6)		360 (91.4)		N/A			N/A		
**History of STD^d^ in P12M^e^**													
	No	594 (94.0)		50 (8.4)		544 (91.6)		8.114 (1)			.004^f^		
	Yes	38 (6.0)		9 (23.7)		29 (76.3)		N/A			N/A		
**History of drug use**													
	No	621 (98.3)		56 (9.1)		565 (90.9)		2.372 (1)			.12^f^		
	Yes	11 (1.7)		3 (27.3)		8 (72.7)		N/A			N/A		
**Psychotropic drug use before last anal intercourse**													
	No	627 (99.2)		56 (8.9)		571 (91.1)		15.285 (1)			.007^b^		
	Yes	5 (0.8)		3 (60.0)		2 (40.0)		N/A			N/A		
**Anal sexual positioning in P6M^g^**													
	Insertive	248 (39.2)		19 (7.7)		229 (92.3)		10.809 (2)			0.004		
	Receptive	214 (33.9)		31 (14.5)		183 (85.5)		N/A			N/A		
	Versatile	170 (26.9)		9 (5.3)		161 (94.7)		N/A			N/A		
**Condom use in the last anal intercourse**													
	No	218 (34.5)		20 (9.2)		198 (90.8)		0.010 (1)			.92		
	Yes	414 (65.5)		39 (9.4)		375 (90.6)		N/A			N/A		
**The number of times participants had anal intercourse in the last week**													
	0	248 (39.2)		21 (8.5)		227 (91.5)		3.319 (2)			.19		
	1	193 (30.5)		24 (12.4)		169 (87.6)		N/A			N/A		
	≥2	191 (30.2)		14 (7.3)		177 (92.7)		N/A			N/A		
**Frequency of condom use in the anal intercourse in P6M**													
	Never	43 (6.8)		2 (4.7)		41 (95.3)		10.056 (2)			.007		
	Sometime	264 (41.8)		36 (13.6)		228 (86.4)		N/A			N/A		
	Every time	325 (51.4)		21 (6.5)		304 (93.5)		N/A			N/A		
**Commercial male sexual**													
	No	526 (83.2)		53 (10.1)		473 (89.9)		2.032 (1)			.15		
	Yes	106 (16.8)		6 (5.7)		100 (94.3)		N/A			N/A		
**Having sex with female**													
	No	562 (88.9)		54 (9.6)		508 (90.4)		0.447 (1)			.50		
	Yes	70 (11.1)		5 (7.1)		65 (92.9)		N/A			N/A		
**Current syphilis infection**													
	Negative	560 (88.6)		43 (7.7)		517 (92.3)		15.943 (1)			<.001		
	Positive	72 (11.4)		16 (22.2)		56 (77.8)		N/A			N/A		
**The number of male sexual partner in P6M**													
	1	192 (30.4)		19 (9.9)		173 (90.1)		0.102 (1)			.75		
	2^+^	440 (69.6)		40 (9.1)		400 (90.9)		N/A			N/A		
**Score of HIV knowledge**													
	<6	43 (6.8)		1 (2.3)		42 (97.7)		1.864 (1)			.17		
	≥6	589 (93.2)		58 (9.8)		531 (90.2)		N/A			N/A		

^a^N/A: not applicable.

^b^Fisher exact test.

^c^Divorced or widowed.

^d^STD: sexually transmitted disease.

^e^P12M: past 12 months.

^f^Continuity correction.

^g^P6M: past 6 months.

### Bivariate Analysis of HIV Infection

A total of 9.3% (59/632) of the participants were HIV-infected and 11.4% (72/632) were syphilis infected. Overall, 2.5% (16/632) had both HIV and syphilis. The bivariate analysis results of factors associated with HIV infection are shown in Table 1.

Participants of Zhuang ethnicity and other ethnicities showed higher HIV infection proportions than those of Han ethnicity (22/148, 14.9% and 7/38, 18.4% vs 30/446, 6.7%, *P*=.002). Participants who used psychotropic drugs in the last anal intercourse had a significantly higher HIV infection rate (3/5, 60.0% vs 56/627, 8.9%, *P*=.007). Moreover, participants with receptive sexual positioning had a significantly higher HIV infection rate than those who had insertive or versatile sex (31/214, 14.5% vs 19/248, 7.7% and 9/170, 5.3%, *P*=.004). In addition, participants who had ever had an STD in the past 12 months had a significantly higher HIV infection rate than those who did not (9/38, 23.7% vs 50/594, 8.4%, *P*=.004). Participants with active syphilis had a higher rate of HIV infection than those without active syphilis (16/72, 22.2% vs 43/560, 7.7%, *P*<.001).

### Multivariable Analysis of Factors Associated With HIV Infection

The results of multivariable logistic regression analyses are demonstrated in Table 2. The Han ethnic group showed a lower likelihood of HIV infection than other ethnic groups (AOR 0.28, 95% CI 0.11-0.71, *P=*.007). HIV infection was associated with receptive anal sexual positioning in the past 6 months (AOR 2.94, 95% CI 1.32-6.53, *P*=.008), inconsistent condom use in the past 6 months (AOR 1.91, 95% CI 1.06-3.45, *P=*.031), and current syphilis infection (AOR 0.38, 95% CI 0.19-0.75, *P=*.005). Although showing statistical significance (AOR 16.70, *P=*.005), psychotropic drug use was not considered a variable that was associated with HIV infection due to the SE of 1.00 and very wide CI of 2.34-119.18.

**Table 2:** Factors associated with HIV infection among young men who have sex with men in Guangxi, China between 2014 and 2015.

Variables		β	SE	Wald test	AOR^a^ (95% CI)	*P* value
**Ethnicity**		N/A^b^	N/A	10.78	N/A	.005
	Han	–1.29	0.48	7.22	0.28 (0.11-0.71)	.007
	Zhuang	–.50	0.50	0.99	0.61 (0.23-1.62)	.32
	Other	N/A	N/A	N/A	Ref^c^	N/A
**Psychotropic drug use before last anal intercourse**						
	No	N/A	N/A	N/A	Ref	N/A
	Yes	2.82	1.00	7.89	16.70 (2.34-119.18)	.005
**Anal sexual positioning in P6M^d^**		N/A	N/A	9.42	N/A	.009
	Insertive	.33	0.44	0.58	1.40 (0.59-3.28)	.45
	Receptive	1.08	0.41	7.00	2.94 (1.32-6.53)	.008
	Both	N/A	N/A	N/A	Ref	N/A
**Frequency of condom use in anal intercourse in P6M**						
	Never or sometimes	.65	0.30	4.65	1.91 (1.06-3.45)	.03
	Every time	N/A	N/A	N/A	Ref	N/A
**Current syphilis infection**						
	No	–.97	0.35	7.73	0.38 (0.19-0.75)	.005
	Yes	N/A	N/A	N/A	Ref	N/A

^a^AOR: adjusted odds ratio.

^b^N/A: not applicable.

^c^Ref: reference.

^d^P6M: past 6 months.

### Sexual Behaviors and HIV-Related Knowledge by Ethnicity

To further understand behavioral and HIV knowledge differences among ethnic groups, we conducted additional analyses. As shown in Table 3, there was a marginal level of statistical difference in HIV-related knowledge between participants of non-Han and those of Han ethnicity (410/446, 91.9% vs 179/186, 96.2%, *P=*.05). The 2 groups did not differ in other variables. We also conducted analyses for 3 ethnic groups: Han, Zhuang, and others. However, 3 groups did not differ regarding scores of HIV knowledge (see Table S1 in Multimedia Appendix 1).

**Table 3:** Sexual behaviors and HIV-related knowledge by ethnicity group among young men who have sex with men.

Variables		Ethnicity, n (%)		Chi-square (*df*)	*P* value
		Han	Non-Han		
**History of drug use**					
	No	438 (98.2)	183 (98.4)	0.000 (1)	≥.99^a^
	Yes	8 (1.8)	3 (1.6)	N/A^b^	N/A
**Psychotropic drug use before last anal intercourse**					
	No	442 (99.1)	185 (99.5)	0.000 (1)	≥.99^a^
	Yes	4 (0.9)	1 (0.5)	N/A	N/A
**Condom use in anal intercourse in P6M^c^**					
	Inconsistent	209 (46.9)	98 (52.7)	1.784 (1)	.18
	Consistent	237 (53.1)	88 (47.3)	N/A	N/A
**The number of times participants had anal intercourse in the last week**					
	0	165 (37.0)	83 (44.6)	4.102 (2)	.13
	1	137 (30.7)	56 (30.1)	N/A	N/A
	2+	144 (32.3)	47 (25.3)	N/A	N/A
**Having sex with female**					
	No	396 (88.8)	163 (87.6)	0.171 (1)	.68
	Yes	50 (11.2)	23 (12.4)	N/A	N/A
**Commercial male sexual**					
	No	365 (81.8)	161 (86.6)	2.095 (1)	.15
	Yes	81 (18.2)	25 (13.4)	N/A	N/A
**Anal sexual positioning in P6M**					
	Insertive	173 (38.8)	75 (40.3)	0.362 (2)	.83
	Receptive	150 (33.6)	64 (34.4)	N/A	N/A
	Versatile	123 (27.6)	47 (25.3)	N/A	N/A
**The number of male sexual partner**					
	1	136 (30.5)	56 (30.1)	0.009 (1)	.92
	2^+^	310 (69.5)	130 (69.9)	N/A	N/A
**Condom use in the last anal intercourse**					
	No	151 (33.9)	67 (36.0)	0.272 (1)	.60
	Yes	295 (66.1)	119 (64.0)	N/A	N/A
**Score of HIV knowledge**					
	<6	36 (8.1)	7 (3.8)	3.842 (1)	.05
	≥6	410 (91.9)	179 (96.2)	N/A	N/A
**Current syphilis infection**					
	No	402 (90.1)	158 (84.9)	3.500 (1)	.06
	Yes	44 (9.9)	28 (15.1)	N/A	N/A

^a^Fisher exact test.

^b^N/A: not applicable.

^c^P6M: past 6 months.

## Discussion

With 9.3% (59/632) of young men who have sex with men being HIV-infected in this sample, this study highlighted that HIV interventions must pay special attention to young men who have sex with men. Previous studies also showed that young men who have sex with men had a higher HIV incidence and were more susceptible to recent HIV infection compared to older men who have sex with men [24]. For example, Mao et al reported an HIV prevalence of 5.4% for young men who have sex with men and 3.6% for men who have sex with men of other age groups in 7 Chinese cities [24]. Zou et al reported a 35% annual increase in the number of university students newly infected with HIV between 2011 and 2015 [28]. This study found that some behaviors and status were associated with HIV infection among young men who have sex with men, such as receptive sexual positioning, a lower frequency of condom use, and current or previous STDs. Therefore, the results of this study could be used to promote interventions targeting young men who have sex with men.

Although preexposure prophylaxis is an effective measure to prevent HIV among men who have sex with men [29], it has not been implemented as a population-level prevention strategy in China; therefore, HIV prevention among young men who have sex with men mainly focuses on behavioral interventions to reduce risk behaviors and promote HIV screening. Safe sex education must be promoted by targeting young men who have sex with men who are hard to reach. Some prevention channels can be added to HIV programs in order to reach young men who have sex with men, such as through peer educators and using social media and web-based platforms. STD prevention must be integrated into HIV programs to increase people’s awareness of these infections. For HIV clinics, STD history must be asked, and screening tests must be conducted for people who screen for HIV to enable early detection and early treatment of possible STDs. Moreover, a stigma reduction campaign is needed to reduce the HIV stigma in communities and among the HIV high-risk population. HIV stigma is not only associated with low HIV knowledge and risk behaviors but also prevents people from receiving HIV tests [27]. Stigma reduction efforts are suggested through various channels, such as social media, HIV and STD prevention programs, sex education targeting young men who have sex with men, and HIV clinics, to further reduce the negative impacts of HIV stigma on HIV prevention and care.

We also found that ethnic minority groups must be more vulnerable to HIV infection. A previous study found that men who have sex with men ethnic minorities had higher rates of HIV infection and differed from Han ethnicity in some sexual behaviors [30]. At the same time, ethnic minorities had significantly higher current syphilis infection rates than the Han group. Moreover, consistent with previous studies [31,32], current syphilis infection may be associated with HIV infection. Therefore, screening and treatment of syphilis could be enhanced and be part of HIV prevention strategies. In addition, the 2 groups did not differ in sexual behavioral variables but differed in drug use history and drug use before last anal sex. Ethnic minority groups in Guangxi were historically disproportionally affected by drugs; it is possible that the differences in HIV infection rates were due to the effects of injection drug use among ethnic groups, although HIV infections caused by injection drug use have shown a decreasing trend in the region in general. Future studies could further explore the impact of injection drug use on ethnic groups and investigate other factors that could possibly be associated with the higher HIV infection rate of non-Han young men who have sex with men in China. Further, a cultural competency component must be considered in local drug and HIV prevention programs to ensure the best programming outcomes, for example, by providing cultural competency training to prevention staff, using multiple languages or dialects on education materials, and having ethnic representatives participate in the design and implementation of these programs.

Consistent with previous studies [31], our findings indicated that the history of STDs in the past 12 months was associated with HIV infection. The possible reasons for this were that ulcerative STDs, including syphilis and herpes simplex virus 2, can cause ulcers or lesions in the genital and rectal mucosa that could become entrances for the HIV virus [33]. Nonulcerative STDs, including chlamydia and gonorrhea, can increase the risk of HIV infection by inducing inflammatory immune cells or potential HIV target cells or causing tiny perturbations to the integrity of genital and rectal mucosa epithelium [34-36]. This finding implied that HIV intervention strategies must pay more attention to people with a history of STDs. In addition, this study also underlined the importance of early diagnosis and treatment of STDs to further reduce the number of new HIV infections.

Unprotected anal intercourse was an established factor associated with HIV infection. In this study, we further found that consistent condom use in the past 6 months was a protective factor for HIV infection. However, 48.6% (307/632) of participants engaged in inconsistent (never or sometimes) condom use during the anal intercourse in the past 6 months in this study. Promoting condom use and behavioral intervention became an urgent need for HIV prevention, especially for this young group, although this group demonstrated generally good HIV knowledge. This study showed generally higher rates of HIV and syphilis infection compared to other men who have sex with men studies in China [16]. One reason was that a considerable proportion of participants engaged in unprotected anal sexual behavior (using a condom in the anal intercourse sometimes or never). Guangxi ranked third highest regarding reported HIV cases [37]. The relatively high number of HIV cases might further aggravate the HIV epidemic in Guangxi [26]. Highly active antiretroviral therap**y** demonstrated benefits not only on HIV treatment but also on HIV prevention [38]. Therefore, early diagnosis and early initiation of treatment are crucial to detect potential HIV infections and prevent HIV transmissions. HIV programs must increase awareness of the risk of unprotected sexual behaviors and promote HIV testing and early treatment in young men who have sex with men.

This study had limitations. The study used a convenient sampling procedure and was conducted in 4 cities in Guangxi. Caution might be needed when generalizing the results to other regions and other populations. Due to the hard-to-reach nature of the population of men who have sex with men, convenient sampling is widely used in HIV research to achieve a desirable sample size. This study could not make a causal inference due to the cross-sectional study design. Future research could adopt more rigorous study designs to test the causal relationships between HIV infection and associated factors among young men who have sex with men. Moreover, although self-report bias and social desirability bias might potentially affect the accuracy of the collected data, especially when sensitive behavioral data are collected, the self-report method is widely used in behavior-related data collection and HIV research and could provide first-hand data. Furthermore, due to the small number of participants who used psychotropic drugs before the last anal intercourse, the study had a large 95% CI (2.46-125.31) for this variable in the multivariate analyses. Future research could expand the recruitment of participants with drug use to further assess this association.

To our knowledge, this was the first study that explored factors associated with HIV infection among young men who have sex with men in Guangxi using a sample with a considerable portion of ethnic minorities. Our findings called attention to the current HIV and syphilis epidemics among young men who have sex with men in Guangxi. Efficient prevention and interventions (ie, condom promotion and preexposure prophylaxis) are urgently needed for this group. Moreover, programs must pay more attention to some groups for HIV prevention, such as ethnic minority groups, people with low education levels, people with STDs, and people who engage in unprotected anal sexual behaviors. HIV stigma reduction and cultural competency are also 2 components that could be adopted in HIV prevention and care efforts to further improve program outcomes.

## Appendix

Multimedia Appendix 1 Table S1. Sexual behaviors and HIV-related knowledge by ethnicity among young men who have sex with men.

## Abbreviations

AOR: adjusted odds ratio
CDC: Centers for Disease Control and Prevention
ELISA: enzyme-linked immunosorbent assay
STD: sexually transmitted disease

## References

[1] Beyrer C, Baral SD, van Griensven F, Goodreau SM, Chariyalertsak S, Wirtz AL, Brookmeyer R (2012). Global epidemiology of HIV infection in men who have sex with men. The Lancet.

[2] (2012). 2012 China AIDS Response Progress Report. Ministry of Health of the People’s Republic of China.

[3] Chinese Center for Disease Control Prevention (2014). Annual Report for HIV/STD/HCV Prevention and Control.

[4] Qin Q, Guo W, Tang W, Mahapatra T, Wang L, Zhang N, Ding Z, Cai C, Cui Y, Sun J (2017). Spatial analysis of the human immunodeficiency virus epidemic among men who have sex with men in China, 2006-2015. Clin Infect Dis.

[5] Zhong F, Liang B, Xu H, Cheng W, Fan L, Han Z, Liang C, Gao K, Mai H, Qin F, Zhao J, Ling L (2014). Increasing HIV and decreasing syphilis prevalence in a context of persistently high unprotected anal intercourse, six consecutive annual surveys among men who have sex with men in Guangzhou, China, 2008 to 2013. PLoS One.

[6] Li X, Wu G, Lu R, Feng L, Fan W, Xiao Y, Sun Z, Zhang H, Xing Hui, Shao Y, Ruan Y (2014). HIV-testing behavior and associated factors among MSM in Chongqing, China: results of 2 consecutive cross-sectional surveys from 2009 to 2010. Medicine (Baltimore).

[7] Yang HT, Tang W, Xiao ZP, Jiang N, Mahapatra T, Huan XP, Yin YP, Wang XL, Chen XS, Fu GF (2014). Worsening epidemic of HIV and syphilis among men who have sex with men in Jiangsu Province, China. Clin Infect Dis.

[8] Mao H, Ma W, Lu H, Wang L, Zheng H, Zhu Y, Peng Z, Yu R, Wang N (2014). High incidence of HIV and syphilis among migrant men who have sex with men in Beijing, China: a prospective cohort study. BMJ Open.

[9] Luo Y, Zhu C, Chen S, Geng Q, Fu R, Li X, Xu K, Cheng J, Ding J (2015). Risk factors for HIV and syphilis infection among male sex workers who have sex with men: a cross-sectional study in Hangzhou, China, 2011. BMJ Open.

[10] Ma X, Zhang Q, He X, Sun W, Yue H, Chen S, Raymond HF, Li Y, Xu M, Du H, McFarland W (2007). Trends in prevalence of HIV, syphilis, hepatitis C, hepatitis B, and sexual risk behavior among men who have sex with men. Results of 3 consecutive respondent-driven sampling surveys in Beijing, 2004 through 2006. J Acquir Immune Defic Syndr.

[11] Ruan Y, Luo F, Jia Y, Li X, Li Q, Liang H, Zhang X, Li D, Shi W, Freeman JM, Vermund SH, Shao Y (2009). Risk factors for syphilis and prevalence of HIV, hepatitis B and C among men who have sex with men in Beijing, China: implications for HIV prevention. AIDS Behav.

[12] Gao L, Zhou F, Li X, Yang Y, Ruan Y, Jin Q (2010). Anal HPV infection in HIV-positive men who have sex with men from China. PLoS One.

[13] Feng L, Ding X, Lu R, Liu J, Sy A, Ouyang L, Pan C, Yi H, Liu H, Xu J, Zhao J (2009). High HIV prevalence detected in 2006 and 2007 among men who have sex with men in China's largest municipality: an alarming epidemic in Chongqing, China. J Acquir Immune Defic Syndr.

[14] Xiao Y, Ding X, Li C, Liu J, Sun J, Jia Y (2009). Prevalence and correlates of HIV and syphilis infections among men who have sex with men in Chongqing Municipality, China. Sex Transm Dis.

[15] Feng LG, Ding XB, Lu RR, Pan CB, Yi HR, Liu HH, Ou YL, Xu J (2008). HIV prevalence and its associated factors among men who have sex with men in Chongqing. Zhonghua Yu Fang Yi Xue Za Zhi.

[16] Wei S, Zhang H, Wang J, Song D, Duan Y, Yu F, She M, Wang M, Zhang H (2013). HIV and syphilis prevalence and associated factors among young men who have sex with men in 4 cities in China. AIDS Behav.

[17] Dong Z, Xu J, Zhang H, Dou Z, Mi G, Ruan Y, Shen L, Min X, Lan G, Li F, Li T, Ning Z, Wu G, She M, Wu Z, China National HIV Prevention Study Group (2014). HIV incidence and risk factors in Chinese young men who have sex with men--a prospective cohort study. PLoS One.

[18] She M, Zhang HB, Wang J, Xu J, Duan YW, Song DD, Wang M, Dong ZX (2012). Investigation of HIV and syphilis infection status and risk sexual behavior among men who have sex with men in four cities of China. Zhonghua Yu Fang Yi Xue Za Zhi.

[19] Lai J, Pan P, Lin Y, Ye L, Xie L, Xie Y, Liang B, Zheng F, Chen R, Wen L, Luo Y, Liang H, Jiang J (2020). A survey on HIV/AIDS-related knowledge, attitudes, risk behaviors, and characteristics of men who have sex with men among university students in Guangxi, China. Biomed Res Int.

[20] Yang Z, Huang Z, Dong Z, Zhang S, Han J, Jin M (2015). Prevalence of high-risky behaviors in transmission of HIV among high school and college student MSM in China: a meta-analysis. BMC Public Health.

[21] Liu JX, Choi K (2006). Experiences of social discrimination among men who have sex with men in Shanghai, China. AIDS Behav.

[22] Feng Y, Wu Z, Detels R (2010). Evolution of men who have sex with men community and experienced stigma among men who have sex with men in Chengdu, China. J Acquir Immune Defic Syndr.

[23] Choi KH, Hudes ES, Steward WT (2008). Social discrimination, concurrent sexual partnerships, and HIV risk among men who have sex with men in Shanghai, China. AIDS Behav.

[24] Mao X, Wang Z, Hu Q, Huang C, Yan H, Wang Z, Lu L, Zhuang M, Chen X, Fu J, Geng W, Jiang Y, Shang H, Xu J (2018). HIV incidence is rapidly increasing with age among young men who have sex with men in China: a multicentre cross-sectional survey. HIV Med.

[25] (2020). Guangxi population data from the seventh national census. People's Daily Online.

[26] Wang X, Lan G, Shen Z, Vermund SH, Zhu Q, Chen Y, Khoshnood K, Wu Z, Tang Z (2014). HIV and syphilis prevalence trends among men who have sex with men in Guangxi, China: yearly cross-sectional surveys, 2008-2012. BMC Infect Dis.

[27] Liao M, Wang M, Shen X, Huang P, Yang X, Hao L, Cox C, Wu P, Tao X, Kang D, Jia Y (2015). Bisexual behaviors, HIV knowledge, and stigmatizing/discriminatory attitudes among men who have sex with men. PLoS One.

[28] Zou H, Tucker JD, Fan S, Xu J, Yu M, Luo Z, Cai W, Grulich AE (2018). Learning about HIV the hard way: HIV among Chinese MSM attending university. The Lancet Infectious Diseases.

[29] Hillis A, Germain J, Hope V, McVeigh J, Van Hout MC (2020). Pre-exposure prophylaxis (PrEP) for HIV prevention among men who have sex with men (MSM): a scoping review on PrEP service delivery and programming. AIDS Behav.

[30] Pan SW, Carpiano RM, Li D, Zhang Z, Schechter MT, Spittal PM, Ruan Y (2018). Ethnicity and HIV vulnerabilities among men who have sex with men in China. AIDS Care.

[31] Xu JJ, Tang WM, Zou HC, Mahapatra T, Hu QH, Fu GF, Wang Z, Lu L, Zhuang MH, Chen X, Fu JH, Yu YQ, Lu JX, Jiang YJ, Geng WQ, Han XX, Shang H (2016). High HIV incidence epidemic among men who have sex with men in china: results from a multi-site cross-sectional study. Infect Dis Poverty.

[32] Jia Z, Huang X, Wu H, Zhang T, Li N, Ding P, Sun Y, Liu Z, Wei F, Zhang H, Jiao Y, Ji Y, Zhang Y, Guo C, Li W, Mou D, Xia W, Li Z, Chen D, Yan H, Chen X, Zhao J, Meyers K, Cohen T, Mayer K, Salomon JA, Lu Z, Dye C (2015). HIV burden in men who have sex with men: a prospective cohort study 2007-2012. Sci Rep.

[33] Dangerfield DT, Smith LR, Williams J, Unger J, Bluthenthal R (2017). Sexual positioning among men who have sex with men: a narrative review. Arch Sex Behav.

[34] Bernstein KT, Marcus JL, Nieri G, Philip SS, Klausner JD (2010). Rectal gonorrhea and chlamydia reinfection is associated with increased risk of HIV seroconversion. J Acquir Immune Defic Syndr.

[35] Cohen CR, Plummer FA, Mugo N, Maclean I, Shen C, Bukusi EA, Irungu E, Sinei S, Bwayo J, Brunham RC (1999). Increased interleukin-10 in the the endocervical secretions of women with non-ulcerative sexually transmitted diseases: a mechanism for enhanced HIV-1 transmission?. AIDS.

[36] Henning TR, Morris M, Ellis S, Kelley K, Phillips C, Ritter J, Jones T, Nachamkin E, Chen CY, Hong J, Kang J, Patton D, McNicholl J, Papp J, Kersh EN (2017). Development of a rectal sexually transmitted infection (STI) Model in Rhesus macaques using Chlamydia trachomatis serovars E and L. J Med Primatol.

[37] Sun X, Yang W, Tang S, Shen M, Wang T, Zhu Q, Shen Z, Tang S, Chen H, Ruan Y, Xiao Y (2020). Declining trend in HIV new infections in Guangxi, China: insights from linking reported HIV/AIDS cases with CD4-at-diagnosis data. BMC Public Health.

[38] Hull M, Lange J, Montaner JS (2014). Treatment as prevention--where next?. Curr HIV/AIDS Rep.

