# Supplementary material for: HIV Prevalence and Risk Factors Among Young Men Who Have Sex With Men in Southwest China: Cross-sectional Questionnaire Study
Source: JMIR Form Res. 2023 Jan 11;7:e37344. doi: 10.2196/37344 (PMC9893886; doi:10.2196/37344)
Supplement: Multimedia Appendix 1 [file formative_v7i1e37344_app1.pdf]

Appendix 1

Table S1. Sexual behaviors and HIV-related knowledge by ethnicity among young men who have sex with men.

	Variables	Ethnicity (%)			χ^2	*p*
		Han	Zhuang	Other		
History of drug use	No	438(98.2)	145(98.0)	38(100.0)	0.242	≥0.05*
	Yes	8(1.8)	3(2.0)	0(0.0)		
Psychotropic drug use before last anal intercourse	No	442(99.1)	147(99.3)	38(100.0)	0.201	≥0.05*
	Yes	4(0.9)	1(0.7)	0(0.0)		
Condom use in anal intercourse in P6M	Inconsistent	209(46.9)	81(54.7)	17(44.7)	2.993	0.224
	Consistent	237(53.1)	67(45.3)	21(55.3)		
The number of times participants had anal intercourse in the last week	0	165(37.0)	62(41.9)	21(55.3)	6.801	0.147
	1	137(30.7)	48(32.4)	8(21.1)		
	2+	144(32.3)	38(25.7)	9(23.7)		
Having sex with female	No	396(88.8)	129(87.2)	37(97.4)	3.226	0.199
	Yes	50(11.2)	19(12.8)	1(2.6)		
Commercial male sexual	No	365(81.8)	127(85.8)	34(89.5)	2.386	0.303
	Yes	81(18.2)	21(14.2)	4(10.5)		
Anal sexual positioning in P6M	Insertive	173(38.8)	65(43.9)	10(26.3)	7.896	0.095
	Receptive	150(33.6)	52(35.1)	12(31.6)		
	Versatile	123(27.6)	31(20.9)	16(42.1)	0.389	0.823
The number of male sexual partner	1	136(30.5)	43(29.1)	13(34.2)		
	2^+	310(69.5)	105(70.9)	25(65.8)	2.263	0.323
Condom use in the last anal intercourse	No	151(33.9)	57(38.5)	10(26.3)		
	Yes	295(66.1)	91(61.5)	28(73.7)		
Score of HIV knowledge	<6	36(8.1)	4(2.7)	3(7.9)	5.128	0.077
	≥6	410(91.9)	144(97.3)	35(92.1)		
Current syphilis infection	No	402(90.1)	126(85.1)	32(84.2)	3.526	0.172
	Yes	44(9.9)	22(14.9)	6(15.8)		

* Fisher's Exact Test was used.
